# Pauperism and Crime

**Published:** 1875-04

**Authors:** 


### PAUPERISM AND CRIME.

The history of legislation regulating pauperism and crime is very instructive. Jails, penitentiaries, prisons, hospitals, &c., are necessary adjuncts to civilized society. If men attack society we must cut them off from general social intercourse, but when thus sequestered, they need not necessarily be treated cruelly and inhumanly. Humanity and Christian charity come in and supplement and soften the effects of the law. Of all classes of the poor and unfortunate, the insane should especially not be treated as brutes. Pauperism is not a crime. Many a man has lived poor beyond anything we ever saw, whose hands were as hard as horn, while his heart was as gentle as a child's. But extreme pauperism certainly tends to vice. That is the philosophy contained in the prayer of Hagar in the wilderness, ' Give me not poverty, lest I steal," &c. The communism in Paris that horrified us a few years ago was the logical result of the socialist principles scattered for years about the faubourgs of Paris. When we make laws that require, by taxation and tithings, one class to support another class, we do both wrong. We take away condescension and kindness on one side and gratitude and friendliness on the other. God has founded human society on the law of love. Love thy God supremely, and thy neighbor as thyself. That is the legislation of the world compacted into one word. In the person of Jesus Christ there was a new enactment of the law of love, and a new foundation for society. On this foundation a solid social structure is entirely practicable

